# Assessment of liver fibrosis by transient elastography and multi-parameters model in young children with chronic hepatitis B virus infection

**DOI:** 10.1186/s12879-022-07142-7

**Published:** 2022-02-18

**Authors:** Haiyan Luo, Songxu Peng, Wenxian Ouyang, Yanfang Tan, Tao Jiang, Lian Tang, Shuangjie Li, Jun Qiu, Changci Zhou

**Affiliations:** 1 Department of Hepatology, Hunan Children’s Hospital, Changsha, 410007 China; 2 Department of Maternal and Child Health, Xiangya School of Public Health, Central South University, Changsha, 410078 China; 3 Pediatrics Research Institute of Hunan Province, Hunan Children’s Hospital, Changsha, 410007 China; 4 Academy of Pediatrics of University of South China, Hengyang, 421001 China

**Keywords:** Chronic hepatitis B, Liver fibrosis, Diagnosis, Transient elastography, Multi-parameter indicators, Children

## Abstract

**Objective:**

This study aimed to compare the diagnostic value of the single or combined applications of transient elastography (TE) and multivariate indicators with biopsy for the detection of liver fibrosis in children caused by chronic hepatitis B (CHB).

**Methods:**

This study included 148 CHB children treated at Hunan Children’s Hospital from January 1st 2015 to December 31st 2018, aged from 0.83 to 14.58 years old. All patients underwent liver biopsy (LB), of which 43 patients underwent TE. Multiple clinical data, including aspartate aminotransferase (AST), alanine aminotransferase (ALT), Platelet (PLT), and HBV-deoxyribonucleic acid (HBV DNA) of all patients were collected. The diagnostic values for CHB of TE and its combinations with these indicators were measured. The patients were classified in two ways: no hepatic fibrosis group (F0) versus fibrosis group (F ≥ 1), and no significant hepatic fibrosis group (F < 2) versus significant hepatic fibrosis group (F ≥ 2). The statistical assessment was performed between groups within each classification to compare the diagnostic value of different parameters.

**Results:**

The operating characteristic area under curve (*AUC*) of liver fibrosis diagnosed by liver stiffness measurement (LSM) which obtained by TE, AST-to-PLT ratio index (APRI), and fibrosis-4 index (FIB-4) were 0.740, 0.701, and 0.651, while the corresponding cut-off values were 5.9 kPa, 0.50, and 0.10, respectively. The *AUC* of significant liver fibrosis diagnosed by LSM, APRI and FIB-4 were 0.849, 0.701, and 0.509, while the corresponding cut-off values were 8.4 kPa, 0.76, and 0.08, respectively. While with the combinations of LSM and APRI, LSM and FIB-4, LSM and APRI and FIB-4, APRI and FIB-4, the *AUC* of significant liver fibrosis were 0.866, 0.855, 0.869, and 0.684, respectively. The *AUC* of significant liver fibrosis diagnosed by the LSM was significantly higher than APRI and FIB-4.

**Conclusions:**

The diagnostic value of transient elastography was better than that of APRI and FIB-4 for CHB children with significant liver fibrosis. In addition, TE also has relatively high application values on the diagnosis of patients with different degrees of liver fibrosis caused by CHB.

## Introduction

Hepatitis B virus (HBV) infection is a widespread public health threat across the world. The hepatitis B serum epidemiological study held by the Chinese Center for Disease Control and Prevention (CDC) in 2014 discovered that the HBsAg detection rate was above 1.26% in children in China [1]. In addition, the age of the individual is the most important factor affecting the outcomes of HBV infection. The earlier the HBV infection occurs, the more likely to develop a chronic HBV infection. As mother-to-child transmission is the dominant way for HBV infection in China, accounting for 30–50% [1], mostly occurring in the perinatal period and transmitting through blood and body fluids of HBV positive mothers, it is necessary to pay more attention to children with chronic hepatitis B (CHB) [1]. CHB can further develop into liver fibrosis, a progressive disease state due to the excessive deposition of extracellular matrix in the liver. Since the failure of prompt treatment may lead to end-stage complications of liver disease such as decompensated liver cirrhosis, liver cancer, and liver failure [2], the control and removal of the etiology of liver fibrosis may be favorable for the disease reversion of liver fibrosis and even cirrhosis [3–5]. In clinical practices, the degree of liver fibrosis is of great significance to guide the treatment of CHB, the monitoring of the disease, and the assessment of the final prognosis of the liver.

Currently, liver biopsy (LB) serves as the gold standard for the diagnosis and staging of liver fibrosis. However, LB is an invasive examination with complications such as right upper quadrant pain, bleeding, infection, pneumothorax, and so on [4]. Furthermore, the repeatability and compliance of operations are poor due to the sampling errors resulting from the limitation of samples, which greatly limits the clinical application, especially for children. The 2015 edition of the Chinese Guidelines for the Prevention and Treatment of Chronic Hepatitis B recommended a non-invasive diagnosis of liver fibrosis for the first time. The main diagnostic methods include fibrosis-4 index (FIB-4) and transient elastography (TE). However, the guideline did not propose non-invasive diagnostic indicators and techniques for liver fibrosis in children [1]. Although there are a lot of researches focused on non-invasive diagnosis models for liver fibrosis in CHB adults, little is about children. A recent study done by Xu et al. found that the liver stiffness measurement (LSM), the aspartate aminotransferase (AST)-platelet (PLT) ratio index (APRI), and FIB-4 were positively correlated with the fibrosis stage in CHB children aged 0–6 years old, and displayed the cut-off values of LSM for significant fibrosis and advanced fibrosis as 5.6 kPa, 6.9 kPa, respectively [6], which was similar to the result of the study by Xu and his colleagues for children under 12 years old but it was different to the results for children above 12 years old [7]. Ulrike Teufel-Schäfer and his colleagues also found that TE showed a good correlation to the histological findings in children with hepatopathy [8]. Due to the small sample size, different operating instruments, limited data, and few studies on CHB in children, it is of great significance to conduct more studies to evaluate the non-invasive diagnostic index of liver fibrosis caused by CHB in children. Our study used samples from Hunan Children’s Hospital to assess the diagnostic value of the single or combined applications of TE and multivariate indicators for liver fibrosis caused by CHB in children.

## Methods

### Patient recruitment

**Fig. 1:**
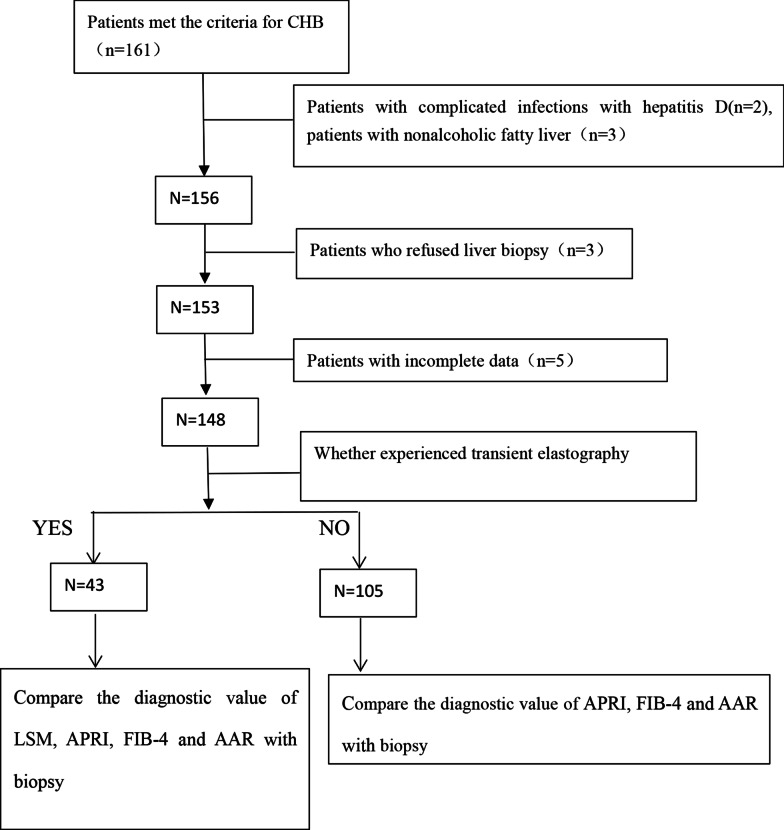
The diagram of patient recruitment

This study recruited 161 hospitalized patients diagnosed with CHB (aged 0–18 years old) at Hunan Children’s Hospital from January 1st 2015 to December 31st 2018. Among them, 2 patients were excluded due to complicated infections with hepatitis D and 3 patients due to nonalcoholic fatty liver. At the same time, 3 patients who refused liver biopsy and 5 patients with incomplete data were also excluded. Finally, 148 eligible patients were enrolled (Fig. 1).They were divided into 5 stages F0, F1, F2, F3, and F4 according to the Scheuer system on pathological staging. After 2016,we introduced TE device and performed TE on 43 eligible patients within two weeks after the LB. They were also divided into 5 stages F0, F1, F2, F3, and F4 according to the Scheuer system on pathological staging.The serologic examinations were performed within two weeks after the LB. The sample size in our study was calculated by the formula as follow: Nsp = (Z_1-α/2_ × √*se*^*[1]*^ × (1 − *se*)/0.1)^2^ = (1.96 × √0.915 × (1–0.915)/0.1)^2^ ≈ 30 [6].This study was approved by the Ethics Committee of Hunan Children’s Hospital (IRB No. HCHLL-2019004), and obtained the formal written informed consent from all the parents and guardians of the participating children. For patients who experienced LB and who underwent both TE and LB, we classified them into two groups in the same ways: no hepatic fibrosis group (F0) and fibrosis group (F ≥ 1), no significant hepatic fibrosis group (F < 2) and significant hepatic fibrosis group (F ≥ 2).

### Detection of HBV-deoxyribonucleic acid (HBV DNA)

Polymerase chain reaction (PCR) and fluorescence probe were used to detect the serum load of HBV DNA with the aid of ankle brachial index (ABI) 7500 instrument (made by Hunan Shengxiang Biological Science and Technology Ltd) and new HBsAg and anti-HBs kit.

### Detection of ALT and AST as well as peripheral platelets

Liver function was monitored by using the Bayer-2400 automatic biochemistry analyzer. The reference ranges of ALT and AST were from 0 to 40 U/L. Peripheral platelets were assayed by the ADVIA2120 automated hematology analyzer and its corollary reagent manufactured by Bayer, Germany.

### Performance of liver biopsy (LB)

The patients were maintained at the supine position or at the slightly lateral recumbent position to determine the puncture with B-ultrasound localization. Percutaneous liver biopsy was performed with Bard needle to obtain the liver tissue with 10% formaldehyde fixation fixed, HE stained and slices routinely prepared. After that, the same senior pathologist should read the slices in a unified manner and observe the pathological changes by the electron microscope to divide the degrees of liver fibrosis into stage F0 to F4 according to the Scheuer scoring system [1].

### Performance of liver stiffness measurement (LSM)

The ultrasonic liver cirrhosis detector produced by Shenzhen Integrated Medical Technology Co, Ltd. was used, which is also a detection technique based on transient elastography. The intercostal space was chosen to measure the hardenability of liver parenchyma. The value of LSM was obtained after the measurement of effective values for ten times. The accuracy of the LSM value included shall be more than 60% with the deviation value being less than 1/3 of the median. All procedures were performed by professionally trained physicians. We performed the examination of transient elastography when children fell asleep. For some children who cannot cooperate with the operation, we have sedated the children with the consent of their parents.

## Hepatic fibrosis scoring system: the Scheuer scoring systems

F0: No fibrosis; F1: Fibrous expansion of portal areas; F2: Periportal fibrosis or portal-portal fibrous septa but intact architecture; F3: Fibrosis with architectural distortion but no obvious cirrhosis; F4: Probable or definite cirrhosis.

## The calculation formula for the non-invasive diagnostic model with multi-parameters

$${\text{APRI }} = \left( {{\text{ AST }}\left( {{\text{ IU}}/{\text{L}}} \right)/{\text{upper level of normal value}}\left( {{\text{IU}}/{\text{L}}} \right) \, /{\text{ PLT }}\left( {{1}0^{{9}} /{\text{L}}} \right) \, } \right) \times {1}00;{\text{ FIB}} - {\text{4 Index }} = \, \left( {{\text{age}}\left( {{\text{year}}} \right) \times {\text{AST }}\left( {{\text{ IU}}/{\text{L}}} \right) \, } \right)/({\text{ PLT }}\left( {{ 1}0^{{9}} /{\text{L }}} \right) \times {\text{ALT}}^{{{1}/{2}}} \left( {{\text{IU}}/{\text{L }}} \right);{\text{AST}} - {\text{to}} - {\text{ALT ratio}}({\text{AAR}}) = {\text{ AST }}\left( {{\text{ IU}}/{\text{L }}} \right)/{\text{ALT }}\left( {{\text{ IU}}/{\text{L }}} \right)$$

### Statistical analysis

SPSS 20.0 software and MedCalc software were used for statistical analysis. Median and inter-quartile range (*IQR*) were calculated for continued non-normal distribution measurement data as APRI, FIB-4 index, AAR, and LSM values. Categorical variables were expressed as the number and percentage of patients. Liver fibrosis stages F0 to F4 were identified by the gold standard for liver fibrosis according to LB.We classified patients into two groups in two ways: no hepatic fibrosis group (F0) and fibrosis group (F ≥ 1), no significant hepatic fibrosis group (F < 2) and significant hepatic fibrosis group (F ≥ 2). Based on the above described classification, the receiver operating characteristic (ROC) curves of the APRI, FIB-4 index, AAR, and LSM values were created and the area under the receiver operating characteristic curve (*AUC*) was calculated within each group. Discrimination and calibration of the model were assessed by *AUC*, the predictive value was classified as low (*AUC* = 0.500–0.700), moderate (*AUC* = 0.700–0.900), or high (*AUC* = 0.900–1.000). Calibration across deciles of risk was evaluated using the Hosmer–Lemeshow goodness-of-fit test. For this test, a *P*-value > 0.05 indicates good calibration [9]. Selecting the point corresponding to the maximum Yorden index was considered as the cut-off point. Then, using MedCalc software analyzed ROC curve to obtain the positive predictive value (*PPV*), negative predictive value (*NPV*), positive likelihood ratio (+ *LR*) and negative likelihood ratio (− *LR*). The ROC contrast test was used to compare ROC curves. Excel software was used to calculate the *Z* value based on the comparison of *AUC* between different groups through the calculation formula (*Z* = (*S*1 − *S*2)/(*SE*1 ^ 2 + *SE*2^2^) ^ 0.5), where *S*1 and *S*2 indicate the area under ROC curve while *SE*1 and *SE*2 signify the corresponding standard error. Then the value of *P* was calculated by the formula *P* value = 1 – NORMSDIST (*Z* value).

Results

Among 148 CHB children who experienced LB, the youngest one was 0.83 years old and the oldest one was 14.58 years old as well as a median of 3.96 years old. In detail, 6 (4.00%) were under 1 year old, 42 (28.40%) were 1–3 years old, 47 (31.80%) were 3–5 years old, 40 (27.00%) were 5–10 years old, and 13 (8.80%) were above 10 years old.There were 28 (18.92%) cases with F0, 94 (63.51%) cases with F1, 19 (12.84%) cases with F2, 5 (3.38%) cases with F3 and 2 (1.35%) cases with F4 (Table1).

**Table 1:** Patient variables

	Variable	Patients^α^ (n = 148)	Patients^β^ (n = 43)
F = 0	n (%)	28 (18.92)	9 (20.93)
	Male, n (%)	20 (71.4)	6 (66.7)
	HBeAg positive, n (%)	28 (100)	9 (100)
	ALT (median, *IQR*, U/L)	35.60 (17.27, 57.28)	19.60 (14.20, 42.37)
	AST (median, *IQR*, U/L)	44.51 (30.99, 65.30)	37.40 (28.70, 54.77)
	PLT (median, *IQR*, 10^9^/L)	305.50 (257.75, 337.00)	310.00 (245.00, 345.50)
	HBVDNA (median, *IQR*, IU/mL)	3.05 × 10^7^ (7.71 × 10^6^, 1.43 × 10^8^)	3.11 × 10^7^ (1.35 × 10^7^, 2.03 × 10^8^)
	Age (median, *IQR*, year)	3.46 (2.60, 5.20)	3.83 (2.71, 5.04)
F = 1	n (%)	94 (63.51)	23 (53.49)
	Male, n (%)	67 (71.3)	16 (69.6)
	HBeAg positive, n (%)	87 (90.6)	21 (91.3)
	ALT(median, *IQR*, U/L)	57.45 (30.93, 96.53)	60.10 (20.00, 89.90)
	AST(median, *IQR*, U/L)	61.34 (42.39, 93.99)	62.10 (26.50, 100.27)
	PLT (median, *IQR*, 10^9^/L)	274.50 (229.75, 323.00)	282.00 (230.00, 326.00)
	HBVDNA (median, *IQR*, IU/mL)	1.49 × 10^7^ (2.56 × 10^6^, 4.01 × 10^7^)	7.60 × 10^6^ (4.56 × 10^6^, 3.00 × 10^7^)
	Age (median, *IQR*, year)	4.40 (2.98, 6.58)	5.83 (3.83, 9.00)
F = 2	n (%)	19 (12.84)	8 (18.60)
	Male, n (%)	16 (84.2)	6 (75.0)
	HBeAg positive, n (%)	16 (84.2)	7 (87.5)
	ALT (median, *IQR*, U/L)	79.00 (52.60, 117.40)	75.40 (45.81, 106.87)
	AST (median, *IQR*, U/L)	78.19 (43.10, 203.90)	77.95 (47.77, 106.87)
	PLT (median, *IQR*, 10^9^/L)	261.00 (226.00, 287.00)	272.50 (248.00, 414.50)
	HBVDNA (median, *IQR*, IU/mL)	7.28 × 10^6^ (9.51 × 10^5^, 7.07 × 10^7^)	7.28 × 10^6^ (1.08 × 10^6^, 1.11 × 10^7^)
	Age (median, *IQR*, year)	3.17 (2.00, 5.08)	4.41 (2.50, 5.33)
F = 3	n (%)	5 (3.38)	2 (4.65)
	Male, n (%)	3 (60)	2 (100)
	HBeAg positive, n (%)	4 (80)	2 (100)
	ALT (median, *IQR*, U/L)	95.20 (60.65, 182.55)	79.50 (95.20)
	AST (median, *IQR*, U/L)	105.30 (73.70, 290.95)	383.80 (105.30)
	PLT (median, *IQR*, 10^9^/L)	289.00 (214.50, 352.00)	289.00 (218.00)
	HBVDNA (median, *IQR*, IU/mL)	4.96 × 10^6^ (4.29 × 10^5^, 5.86 × 10^7^)	100.00 (4.96 × 10^6^)
	Age (median, *IQR, *year)	3.67(2.75, 8.16)	4.08 (3.25)
F = 4	n (%)	2 (1.35)	1 (2.33)
	Male, n (%)	1 (50)	1 (100)
	HBeAg positive, n (%)	1 (50)	0 (0)
	ALT (median, *IQR*, U/L)	134.20 (324.24)	324.24
	AST (median, *IQR*, U/L)	234.90 (548.33)	548.33
	PLT (median, *IQR, *10^9^/L)	164 (217)	217
	HBVDNA (median, *IQR*, IU/mL)	1.38 × 105(8.43 × 10^6^)	8.43 × 10^6^
	Age (median, *IQR*, year)	1.42 (1.17)	1.17

*IQR* inter-quartile range, *ALT* alanine aminotransferase, *AST* aspartate aminotransferase, *ALP* alkaline phosphatase, *PLT* platelet, *HBeAg* hepatitis B e-antigen

α:Patients underwent liver stiffness measurement (LSM) by TE and liver biopsy

β:Patients just underwent liver biopsy

Among the 43 patients who underwent both TE and LB, the youngest was 1.08 years old, the oldest was 14.58 years old. 9 (21.00%) were 1–3 years old, 14 (32.50%) were 3–5 years old, 16 (37.20%) were 5–10 years old, and 4 (9.30%) were above 10 years old. There were 9 (20.93%) cases with F0, 23 (53.49%) cases with F1, 8 (18.60%) cases with F2, 2 (4.65%) cases with F3 and 1 (2.33%) case with F4 (Table1).

In terms of the disease course, the longest case reached 13 years while the shortest last for 1 week with a median value of 1.08 years. There were 107 males and 41 females, accounting for 72.30% and 27.70%, respectively. The HBeAg positive cases were 136, making up for 91.89%. Mothers of 130 (87.84%) patients had a history of hepatitis B virus infection.The detailed information was shown in Table 1.

**Fig. 2:**
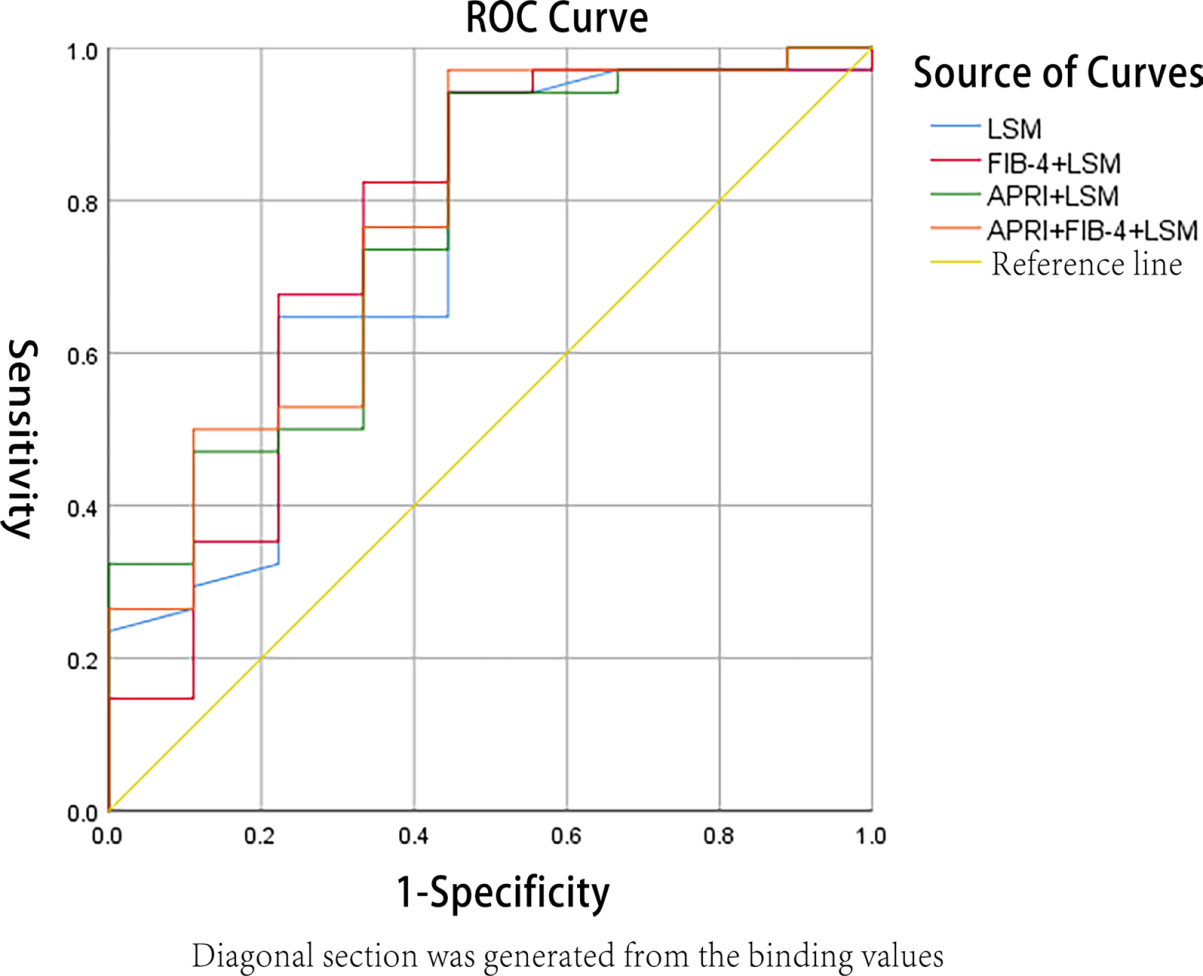
The diagnostic values of LSM and combination of LSM, FIB-4 and APRI for liver fibrosis. *AUC*s of LSM for the diagnosis of liver fibrosis was 0.740 (95% *CI*: 0.543–0.938); Of FIB-4 combined LSM was 0.758 (95% *CI*: 0.555–0.961); Of APRI combined LSM was 0.761 (95% *CI*: 0.580–0.942); Of combination of LSM, APRI and FIB-4 was 0.771 (95% *CI*: 0.580–0.942)

**Fig. 3:**
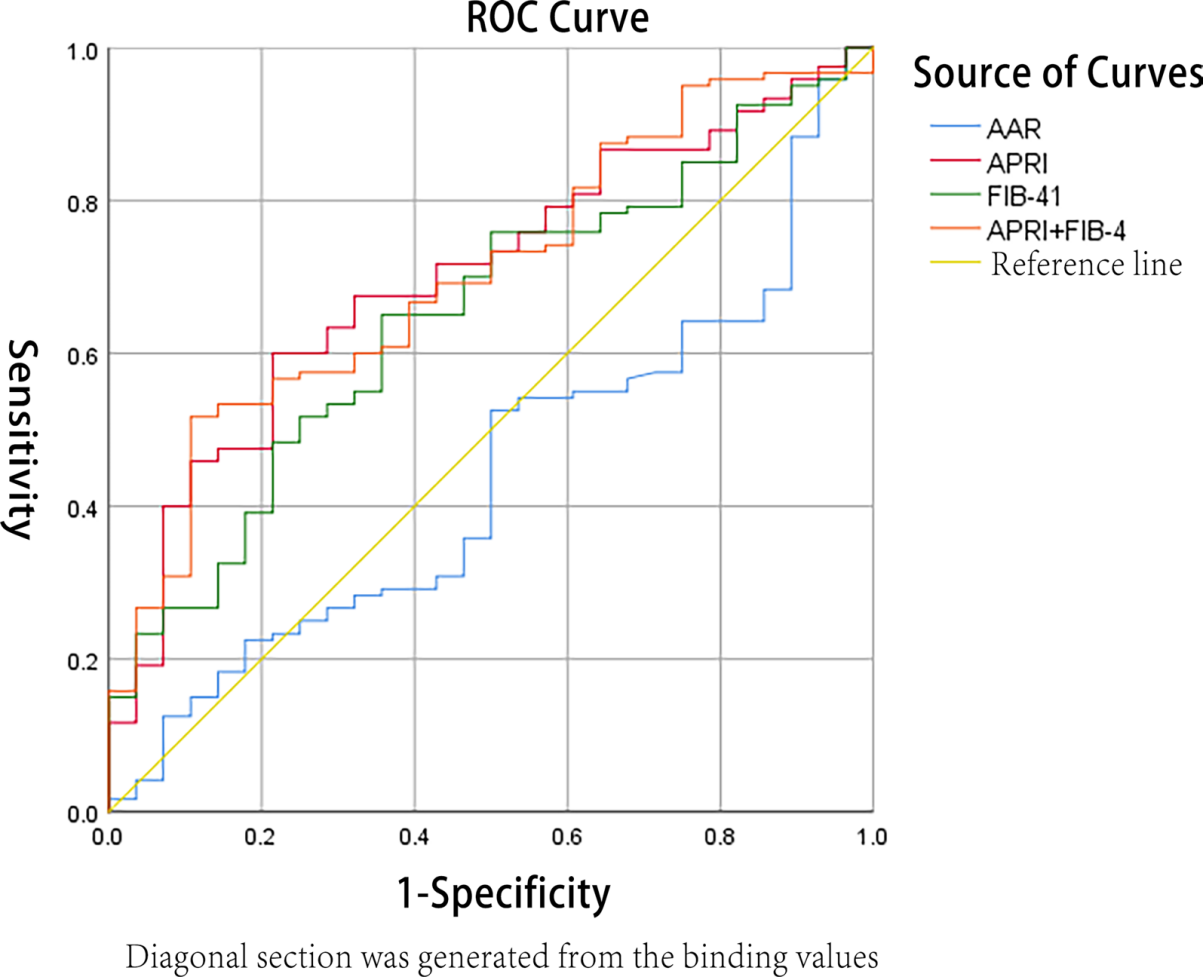
The diagnostic values of APRI, FIB-4 and AAR for liver fibrosis. *AUC*s of APRI for the diagnosis of liver fibrosis was 0.701 (95% *CI*: 0.603–0.800); Of FIB-4 was 0.651 (95% *CI*: 0.546–0.755); Of AAR was 0.440 (95% *CI*: 0.328–0.552); Of the combination of APRI and FIB-4 was 0.703 (95% *CI*: 0.605–0.801)

Based on LSM value, *AUC* for the diagnosis of liver fibrosis in CHB patients was 0.740 (95% *CI*: 0.543–0.938), and the cut-off value, sensitivity (*Se*), specificity (*Sp*), positive predictive value (*PPV*), negative predictive value (*NPV*), positive likelihood ratio (+ *LR*) and negative likelihood ratio (− *LR*) for fibrosis were 5.9 kPa, 94.12%, 55.56%, 88.90%, 71.40%, 2.12 and 0.11 respectively. *AUC* for the diagnosis of liver fibrosis in CHB patients based on APRI value was 0.701 (95% *CI*: 0.603–0.800), the cut-off value, *Se*, and *Sp* for fibrosis were 0.50, 60.00%, and 78.57%, respectively. Based on the FIB-4 index, *AUC* for the diagnosis of liver fibrosis was 0.651 (95% *CI*: 0.546–0.755), the cut-off value, *Se*, and *Sp* for fibrosis were 0.10, 65.00%, and 64.29%, respectively. As for AAR value, *AUC* for the diagnosis of liver fibrosis was 0.440 (95% *CI*: 0.328–0.552), the cut-off value, *Se*, and *Sp* for fibrosis were 0.90, 35.83%, and 85.71%, respectively. *AUC* for the diagnosis of liver fibrosis in CHB patients based on a combination of LSM, APRI and FIB-4 was 0.771 (95% *CI*: 0.580–0.942), which meant that the combination had a good discrimination of liver fibrosis, and a result of the Hosmer-Lemeshow goodness-of-fit test for logistic regression confirmed that the combination was well calibrated (*χ*^*2*^ = 0.170, *P* = 0.264). The *AUC* value (0.740) of liver fibrosis diagnosed by LSM value was higher than that of APRI value (0.701), the differences of which showed no statistical significance (Z was equal to 0.346 while *P* was equal to 0.364). The *AUC* value (0.740) of liver fibrosis diagnosed by LSM value was higher than that of FIB-4 value (0.651), the differences of which showed no statistical significance (*Z* was equal to 0.780 while *P* was equal to 0.218). The *AUC* value (0.771) of liver fibrosis diagnosed by combining LSM and APRI as well as FIB-4 index was higher than that of LSM value (0.740), which showed no significant difference (*Z* was equal to 0.225 while *P* was equal to 0.411). The values of *Se*, *Sp*, *PPV*, *NPV*, + *LR* and − *LR* in the diagnosis of liver fibrosis with biochemistry indicators were listed, all of which were shown in the following Table2, Figs. 2, and 3.

**Table 2:** The area under ROC curve and its relevant parameters of liver fibrosis diagnosed by four non-invasive diagnostic indicators single or in combination

Parameters	n	*AUC*	*P*	95% *CI*	Cut-off	Se (%)	Sp (%)	PPV (%)	NPV (%)	+ LR	− LR	*P*	*X*^*2*^
APRI	148	0.701	0.001	0.603–0.800	0.50	60.00	78.57	92.30	31.40	2.80	0.51		
AAR	148	0.440	0.326	0.328–0.552	0.90	35.83	85.71	91.50	23.80	2.51	0.75		
FIB-4	148	0.651	0.013	0.546–0.755	0.10	65.00	64.29	88.60	30.00	1.82	0.54		
APRI + FIB-4	148	0.703	0.001	0.605–0.801	0.80	51.67	89.29	95.40	30.10	4.82	0.54	0.560	6.788
LSM	43	0.740①	0.028	0.543–0.938	5.9 kPa	94.12	55.56	88.90	71.40	2.12	0.11		
APRI + LSM	43	0.761	0.017	0.580–0.942	0.57	94.12	55.56	88.90	71.40	2.12	0.11	0.636	6.104
FIB-4 + LSM	43	0.758	0.018	0.555–0.961	0.58	94.12	55.56	88.90	71.40	2.12	0.11	0.384	8.529
LSM + APRI + FIB-4	43	0.771	0.017	0.580–0.942	0.53	97.06	55.56	89.20	83.30	2.18	0.05	0.264	0.170

①Compared with APRI, *Z* = 0.346, *P* = 0.364 ①compared with FIB-4, *Z* = 0.780, *P* = 0.218; ①compared with LSM + APRI + FIB-4, *Z* = 0.225, *P* = 0.411

**Fig. 4:**
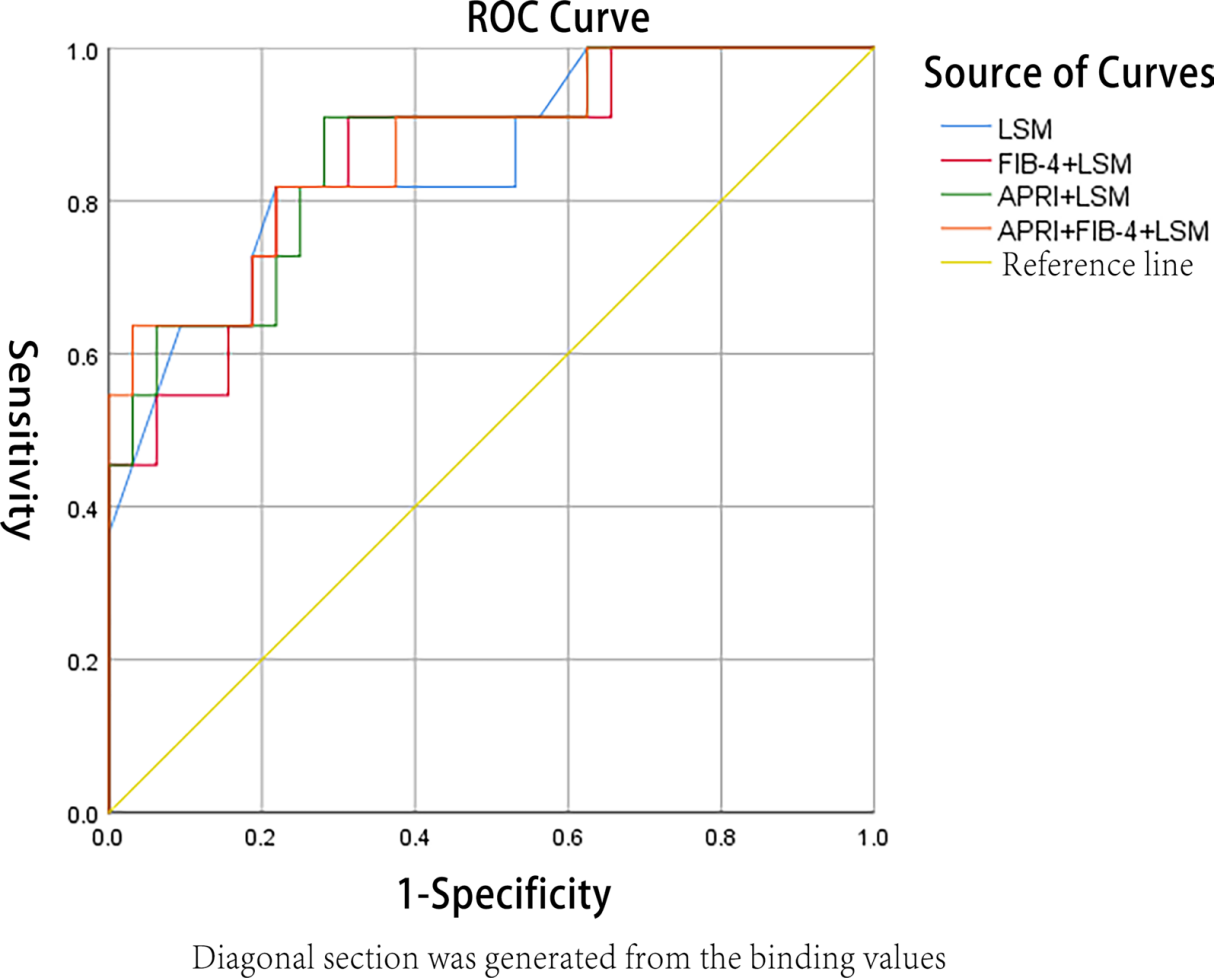
The diagnostic values of LSM and a combination of LSM, FIB-4 and APRI for significant liver fibrosis. *AUC*s of LSM for the diagnosis of significant liver fibrosis was 0.849 (95% *CI*: 0.713–  0.986); Of FIB-4 combined LSM was 0.855 (95% *CI*: 0.723–0.986); Of APRI combined LSM was 0.866 (95% *CI*: 0.740–0.993); Of combination of LSM, APRI and FIB-4 was 0.869 (95% *CI*: 0.741–0.998)

**Fig. 5:**
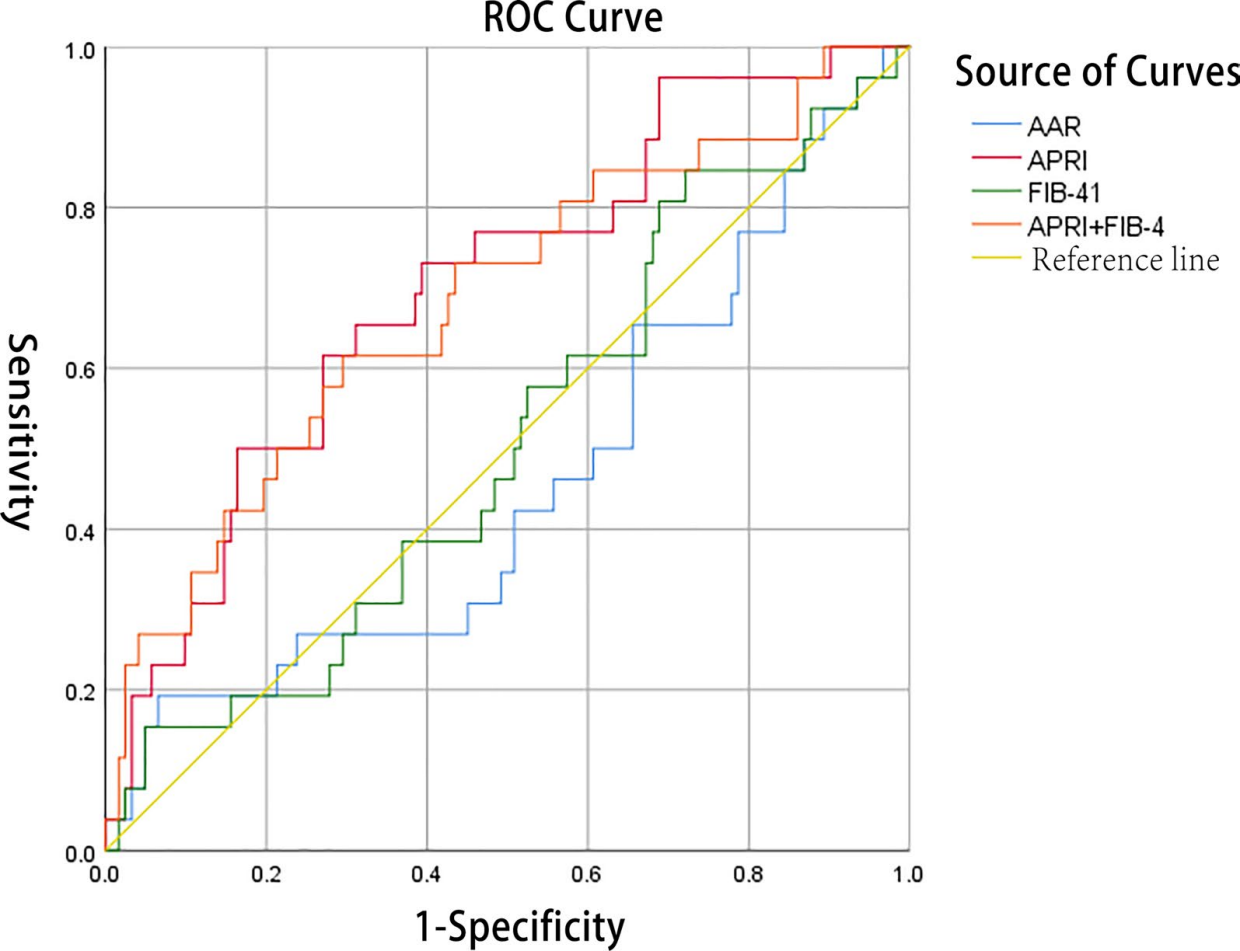
The diagnostic values of APRI, FIB-4 and AAR for significant liver fibrosis. *AUC*s of APRI for the diagnosis of significant liver fibrosis was 0.701 (95% *CI*: 0.591–0.810); Of FIB-4 was 0.509 (95% *CI*: 0.388–0.630); Of AAR was 0.458 (95% *CI*: 0.329–0.586); Of the combination of APRI and FIB-4 was 0.684 (95% *CI*: 0.565–0.802)

*AUC* for the diagnosis of significant liver fibrosis in CHB patients based on LSM value was 0.849 (95% CI: 0.713–0.986), and the cut-off value, *Se, Sp*, *PPV*, *NPV*, + *LR* and − *LR* for significant liver fibrosis were 8.4 kPa, 81.82%, 78.12%, 56.20%, 92.60%, 3.74 and 0.23 respectively. *AUC* for the diagnosis of significant liver fibrosis in CHB patients based on APRI value was 0.701 (95% *CI*: 0.591–0.810), the cut-off value, *Se*, and *Sp* for fibrosis were 0.76, 61.54%, and 72.95%, respectively. Based on the FIB-4 index, *AUC* for the diagnosis of significant liver fibrosis was 0.509 (95% *CI*: 0.388–0.630), and the cut-off value, *Se*, and *Sp* for fibrosis were 0.08, 84.62%, and 27.87%, respectively. As for AAR value, *AUC* for the diagnosis of significant liver fibrosis was 0.458 (95% CI: 0.329–0.586), the cut-off value, *Se*, and *Sp* for fibrosis were 1.13, 69.23%, and 48.36%, respectively. *AUC* for the diagnosis of significant liver fibrosis in CHB patients based on a combination of LSM, APRI and FIB-4 was 0.869 (95% *CI*: 0.741–0.998), which means that the combination had a good discrimination of significant liver fibrosis, and a result of the Hosmer-Lemeshow goodness-of-fit test for logistic regression confirmed that the combination was well calibrated (*χ*^*2*^ = 4.619, *P* = 0.797). The *AUC* value (0.849) of significant liver fibrosis based on LSM value was higher than that of APRI value (0.701), the differences of which reached statistical significance (*Z* were equal to 1.650 while *P* were equal to 0.049). The *AUC* value (0.849) of significant liver fibrosis based on LSM value was higher than that of FIB-4 (0.509),the differences of which reached statistical significance (Z were equal to 3.636 respectively while *P* were equal to 0.000). The *AUC* value (0.869) of significant liver fibrosis by the combination of LSM value and APRI value as well as FIB-4 index was higher than that of LSM value (0.849). There were no significant statistical differences in this case when *Z* was equal to 0.208 and *P* was equal to 0.418. The values of *Se*, *Sp*, *PPV*, *NPV*, + *LR*, and − *LR* in the diagnosis of significant liver fibrosis with biochemistry indicators were listed. They were clearly shown in the following Table 3, Figs. 4, and 5.

**Table 3:** The area under ROC curve and its relevant parameters of significant liver fibrosis diagnosed by four non-invasive diagnostic indicators single or in combination

Parameters	n	*AUC*	*P*	95% *CI*	Cut-off	Se (%)	Sp (%)	PPV (%)	NPV (%)	+ LR	− LR	*P*	*X*^*2*^
APRI	148	0.701	0.001	0.591–0.810	0.76	61.54	72.95	32.70	89.90	2.28	0.53		
AAR	148	0.458	0.500	0.329–0.586	1.13	69.23	48.36	22.20	88.10	1.45	0.76		
FIB-4	148	0.509②	0.884	0.388–0.630	0.08	84.62	27.87	20.00	89.50	1.17	0.55		
APRI + FIB-4	148	0.684	0.003	0.565–0.802	0.16	61.54	70.49	30.80	89.60	2.09	0.55	0.970	6.698
LSM	43	0.849①	0.001	0.713–0.986	8.4 kPa	81.82	78.12	56.20	92.60	3.74	0.23		
APRI + LSM	43	0.866	0.000	0.740–0.993	0.15	90.91	71.87	52.60	95.80	3.23	0.13	0.634	6.122
FIB-4 + LSM	43	0.855	0.001	0.723–0.986	0.24	81.82	78.12	56.20	92.60	3.74	0.23	0.570	6.698
APRI + FIB-4 + LSM	43	0.869	0.000	0.741–0.998	0.47	63.64	96.87	87.50	88.60	20.36	0.38	0.797	4.619

①Compared with APRI, Z = 1.650, *P* = 0.049; ①compared with FIB-4, *Z* = 3.636, *P* = 0.000; ①compared with APRI + FIB-4 + LSM, *Z* = 0.208, *P* = 0.418

## Discussion

TE is a relatively mature non-invasive diagnostic technique for liver fibrosis. The ultrasonic liver cirrhosis detector used in our study is an operation instrument based on the principle of TE. It has the same advantages of noninvasiveness and good repetitiveness as Fibroscan and Fibrotouch. This study showed that TE and APRI were effective in diagnosing young CHB patients with liver fibrosis, with an *AUC* value above 0.700. TE and APRI could perform well in differentiating the stages of liver fibrosis in children with CHB. The results of LSM and APRI in the diagnosis of CHB patients with significant liver fibrosis were good, with the *AUC* values all being more than 0.700. It was shown that TE can better differentiate patients with significant liver fibrosis than APRI. The diagnostic value of TE was better than that of APRI and FIB-4 for CHB children with significant liver fibrosis. The combination of TE and multi-parameter indicators failed to significantly improve their abilities in the diagnosis of varying degrees of liver fibrosis in our study.

Our results showed that TE was superior to APRI and FIB-4 index in the diagnosis of hepatic fibrosis in terms of value, which was consistent with the results obtained by Orasan et al. [10] and Guo Feng et al. [11]. Teshale and colleagues found that the FIB-4 index can better differentiate mild liver fibrosis from significant liver fibrosis in adults with CHB [12]. However, results from our research suggested that the FIB-4 index failed to differentiate mild liver fibrosis from significant liver fibrosis in children with CHB. A study by Wang et al. showed that age has a bearing on the accuracy of the FIB-4 index in the diagnosis of significant liver fibrosis [13]. As thus, there is a need to take the effect of age into account in the application of the FIB-4 index. The AAR values failed to perform well in the diagnosis of CHB patients with varying degrees of liver fibrosis, with *AUC* values all being less than 0.700, which was in accord with the findings of Eminler et al. [14]. However, there is still space for improvement with regard to the considerations of the test results influenced by the sample size and the cooperative degree of the children in the process of the tests and other factors. There is also a need for us to verify the results with a larger sample size so as to reduce the influence of external factors.

The *AUC* values were calculated to analyze the diagnostic value of TE and its combination of different parameters on liver fibrosis. The results showed that the combination of TE and multi-parameter indicators failed to significantly improve their abilities in the diagnosis of varying degrees of liver fibrosis, which was complied with the results by Zeng et al. [15]. However, it was also found that the combination of APRI and FIB-4 index can improve the diagnostic capacity for liver fibrosis of patients with CHB [16]. The sample size, age and other factors of the study should be taken into account.

Our results showed that the best cut-off values of LSM for the diagnosis of liver fibrosis and significant liver fibrosis were 5.9 kPa and 8.4 kPa, respectively. In this case, the sensitivities were 94.12% and 81.82%, and specificities were 55.56% and 78.12%, respectively. Goyal R. found that adults with liver fibrosis in stage F2, F3 can be excluded by FibroScan measured LSM value < 6 kPa, and LSM value > 9 kPa indicated that the degree of liver fibrosis reaches stage F2 or F3, and its sensitivity and specificity were above 90% [17]. Chang P. E. and others found that the optimal cut-off for LSM diagnostic significant liver fibrosis (F ≥ 2) in adults was 9.0 kPa, whose sensitivity and specificity were 67.4% and 75.4%, respectively [18]. And a recent study found that the cut-off values (specificity,sensitivity) for significant fibrosis and advanced fibrosis were 5.6 kPa (75.7%, 67.4%), 6.9 kPa (91.5%, 81.3%) respectively in CHB children aged 0–6 years [6]. The research of Xu ZQ and colleagues showed that the value of LSM increased with age, as the cut-off values for significant liver fibrosis (F ≥ 2) and progressive liver fibrosis (F ≥ 3) in children under 12 years old were 5.8 and 7.0 kPa, respectively, and they were 6.6 and 8.0 kPa for children above 12 years old [7]. The best cut-off values of LSM in these researches were somewhat different. As for the factors influencing the LSM values, Lin et al. [19] found that the LSM values of CHB patients may be in connection with the level of AST, ALT, total bilirubin, and albumin. The studies conducted by Piscaglia F. and other scholars [20] found that most detectors with TE as the principle were in intermediate consistency with the results of Fibroscan, indicating that the general use of Fibroscan thresholds for defining the stage of liver fibrosis in all new machines was not feasible. So, the effects of operating instruments, ALT, AST, age of the patients and other clinical parameters, as well as the cooperative degree of children in the process of operation on TE should be considered. The influence factors of TE technology need to be studied by more data. The sample size needs to be further enlarged to explore the cut-off value for defining the stages of CHB patients with liver fibrosis by TE.

This study had several limitations. First, the number of patients, especially the sample size of patients with the F3-F4 fibrosis stage was small, and the number of patients we selected who were performed TE was small.Second, in the practical operation of TE testing, the difficulty to cooperate with young children and the impact of related factors such as the age, BMI and laboratory indicators of the children could interfere with the accuracy.Third, there are five scoring systems to evaluate the degree of liver fibrosis. In this study, we used Scheuer scoring systems, which might not be the most accurate one. Further studies to compare the accuracy of the different scoring systems are necessary. Future studies need to utilize larger sample sizes and stratify the patients according to their age, BMI and laboratory indicators.

## Conclusion

Transient elastography performed better than APRI and FIB-4 index in the diagnosis of young CHB patients with significant liver fibrosis, with a relatively high application value for the clinical diagnosis of CHB patients with varying degrees of liver fibrosis. However, we need to consider the impact of relevant clinical factors on the test results in the process of application.

## Data Availability

The data and materials used and analyzed in this study are available from corresponding author on reasonable request.

## Abbreviations

ALT: Alanine aminotransferase
AST: Aspartate aminotransferase
*AUC*: The area under receiver operating characteristic
APRI: Aspartate aminotransferase (AST) -to- platelet (PLT) ratio index
AAR: AST-to-ALT ratio
CHB: Chronic hepatitis B
HBV: Hepatitis B virus
LB: Liver biopsy
LSM: Liver stiffness measurement
*−** LR*: Negative likelihood ratio
+ *LR*: Positive likelihood ratio
*NPV*: Negative predictive value
*PLT*: Platelet
*PPV*: Positive predictive value
*ROC*: Receiver operating characteristic
*Se*: Sensitivity
*Sp*: Specificity
